# Accuracy of digital model generated from CT data with metal artifact reduction algorithm

**DOI:** 10.1038/s41598-021-89298-x

**Published:** 2021-05-14

**Authors:** Chena Lee, Ari Lee, Yoon Joo Choi, Kug Jin Jeon, Young Hyun Kim, Sang-Sun Han

**Affiliations:** Department of Oral and Maxillofacial Radiology, Yonsei University College of Dentistry, 50-1 Yonsei-ro Seodaemun-gu, Seoul, 03722 Korea

**Keywords:** Dental diseases, Materials for devices

## Abstract

This study investigated whether metal artifact reduction (MAR) applied computed tomography (CT) scans could be used to generate precise digital models and explored possible correlations between the amount of metal artifact and model accuracy. Thirty maxillofacial CT scans were randomly selected and a MAR algorithm was applied. By subtracting the original and MAR-applied CT images, the amount of metal artifact was quantified. Digital models were generated from the original and the MAR-applied CT data. Paired digital models were superimposed and shape deviation in planar surface was measured at 10 points in 4 planes. Statistical analyses were performed to compare deviations and to assess correlations between the amount of artifact and deviation. The MAR algorithm reduced metal artifact in all cases. The overall mean deviation of the MAR-applied models was 0.0868 mm, with no significant difference according to the reference plane. The amount of artifact did not significantly influence the accuracy of the digital models. MAR-applied CT is a convenient source for digital modeling with clinically acceptable accuracy. The MAR algorithm can be used regardless of the amount of metal artifact, which are generated by dental prostheses, for the quick and convenient manipulation of dental digital models.

## Introduction

Three-dimensional (3D) digital models produced from computed tomography (CT) data have played an increasingly important role in patient education, planning, simulation, and navigation surgery in the oral and maxillofacial region. Generating precise and fine digital models in standard tessellation language (STL) from Digital Imaging and Communications in Medicine data (DICOM) has been a useful technique in maxillofacial region^1,2^. However, dental prostheses cause metal artifact in images, preventing the development of smooth and satisfactory 3D digital models. Metal artifact corrupt the entire axial image section that includes the prosthesis. The artifact also appears as spicule-shaped area that disrupt generated digital models^3^. Previous studies have attempted to solve this problem by integrating CT and other digital dental scan data^4^. However, doing so requires additional work and a registration program. If a dental scanner and registration system are not accessible, the spicules due to metal artifact around the dental arch in a digital model should be removed manually on the computer-aided design (CAD) software.

Metal artifact reduction (MAR) algorithms can be applied to CT images affected with metal artifact. These algorithms have advanced considerably, and effectively recover images corrupted with metal artifact. Thus, digital model in STL format can be generated as smooth without spicules when it is based on the CT data applied with MAR algorithm. In this way, digital model producing is much easier and simpler to use for surgical simulations or patient education. Furthermore, it is advantageous for clinicians, because CT data with MAR algorithm enables them to save time and to avoid the laborious work needed to manipulate and refine the digital models before 3D printing.

However, the MAR process alters the projection data, which are the primary data stored on the workstation of the CT console. This means that the MAR algorithm may modify the non-artifact image data while processing the artifact area. Basically, the algorithm identifies and segments the corrupted data by metal artifact. Then, an appropriate estimate of the corrupted data is predicted based on the uncorrupted data of the original projection. The estimated data is interpolated with the original data to produce metal artifact removed data. After repeated execution of the overall process, the finalized projection data form an axial CT image^5–7^. This process effectively removes data that have been corrupted by metal artifact, but may lead to minor deformations in the entire CT data^8^.

The accuracy of MAR-applied CT image has been studied^9–11^. However, no research has yet analyzed its influence on STL formatted digital models produced from MAR-applied CT data. Since the MAR algorithm may damage the overall CT image, a study of the accuracy of the digital models produced with MAR-applied CT data is necessary. Digital models used for the oral and maxillofacial region require a certain level of accuracy due to the sophisticated nature of the anatomy. In addition, considering the various numbers and locations of dental prostheses, the influence of different amount of metal artifact on the digital model accuracy, produced from MAR-applied CT data should be studied.

Therefore, this study was conducted to verify the accuracy of digital dental models generated from CT data to which a MAR algorithm was applied. Also, the influence of the metal artifact amount on the digital model accuracy was explored to determine clinical applicability of MAR algorithm for the model generation.

## Materials and methods

**Figure 1:**
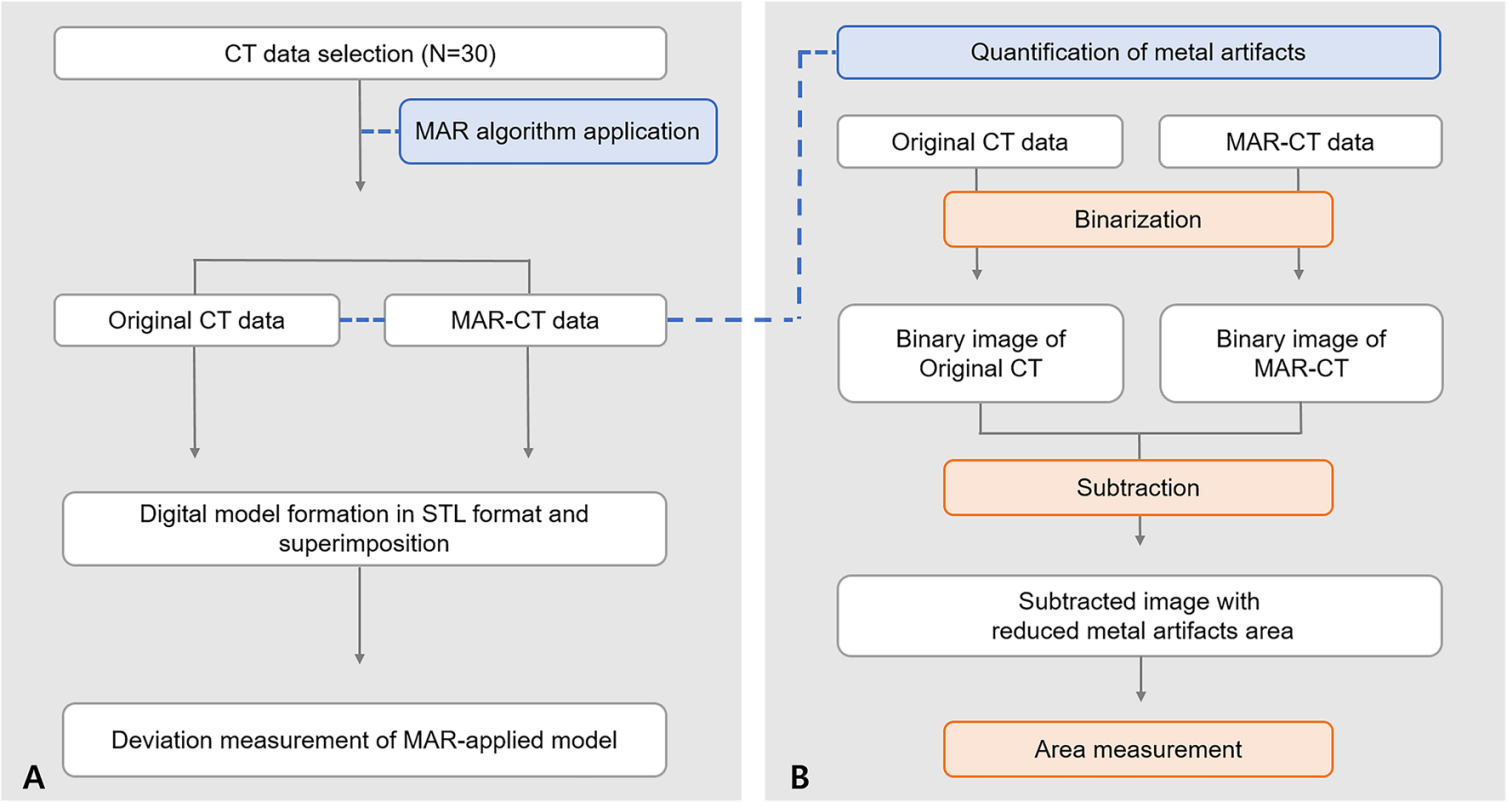
Schematic workflow of (**A**) the overall study procedure and (**B**) the metal artifact quantification procedure. *MAR* metal artifact reduction; *STL* standard tessellation language.

This study was approved by the Institutional Review Board (IRB) of Yonsei University Dental Hospital (No. 2-2020-0025) and was carried out in accordance with relevant guidelines and ethical regulations. Due to the retrospective aspect of this study, informed consent was waived by the by the IRB of Yonsei University Dental Hospital because of the retrospective nature of the study. From February to March 2019, maxillofacial CT data from 30 patients, obtained for the purpose of dental care, were randomly selected. All CT data were obtained with an Optima 520 device (GE Healthcare, Chicago, IL, USA) under the following exposure conditions: 120 kVp, 70 mA, and 0.9 pitch. Overall study procedure was presented Fig. 1.

### MAR application and digital model formation

For the selected CT data (original CT data), a paired dataset was obtained with a MAR algorithm applied (MAR-CT data) via the CT console of the workstation (GE Healthcare, Milwaukee, WI, USA). In both the original and the MAR-applied CT data, jaw bone segmentation was performed based on the threshold of 160 to 3071 Hounsfield units (HU). The segmented jaw bone was converted into an 3D model in STL format. The segmentation and conversion processes were performed on the AW Server (GE Healthcare, Milwaukee, WI, USA). The 3D digital model from the original CT data was referred to as the original model and that from the MAR-CT data was referred to as the MAR-applied model.

### Deviation measurement

The MAR-applied model was superimposed on the original model in Geomagic Control X (3D Systems, Cary, NC, USA). Both models were based on the same CT data with the same X, Y and Z coordination and additional registration was not required. The orientation of the model was aligned as the occlusal plane aligned parallel to the floor plane. Then 4 reference planes, 2 axial and 2 coronal planes, were chosen. Each reference plane included a specific anatomical structure, while running parallel to the floor plane. The anatomical points in the reference planes were as follows.

1. Axial maxilla plane: A-point
2. Axial mandible plane: Right mental foramen
3. Coronal right plane: The lowest point of the right sigmoid notch
4. Coronal left plane: The lowest point of the left sigmoid notch

**Figure 2:**
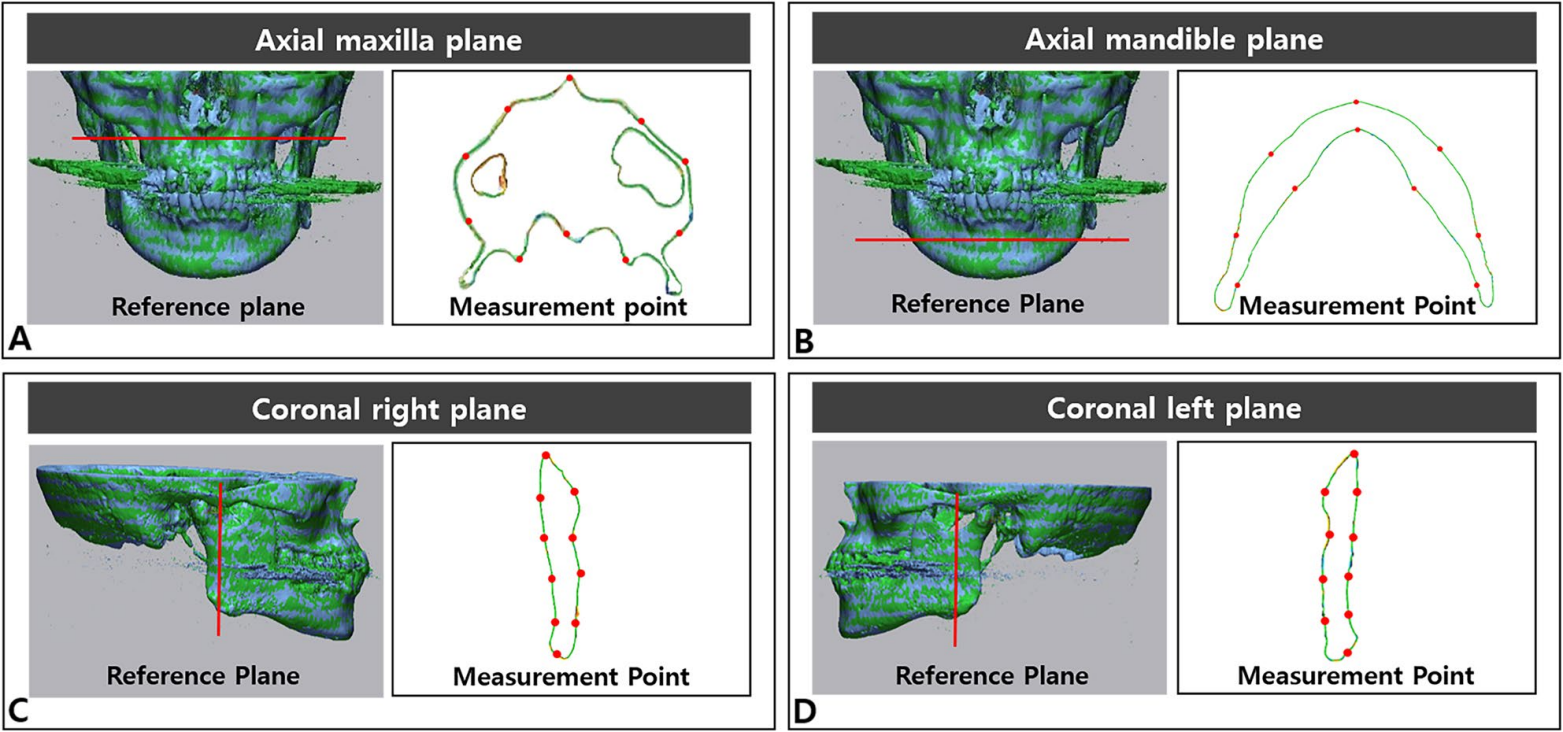
Determination of reference planes and measurement points on superimposed digital models for deviation assessment. The red line indicates a reference plane and the red dot indicates a point of measurement. The anatomical point of each reference plane is as follows. (**A**) A-point, (**B**) right mental foramen, (**C**) the lowest point of the right sigmoid notch, and (**D**) the lowest point of the left sigmoid notch. The starting point for the measurement points is the most superior or anterior point, with subsequent points automatically generated at the same interval.

On each reference plane, 10 points at the same interval were established automatically by the software, and the starting point was always the most anterior point for the axial planes and the highest point for the coronal planes. The reference planes and measurement points were described in Fig. 2. The deviation of MAR-applied model compared to the original model on the outer shape at determined point was measured automatically by the software. This value was defined as shape deviation in planar surface. Two observers performed the same measurement process twice with time interval.

### Metal artifact quantification

The amount of metal artifact was quantified using the ImageJ software version 1.8.0 (National Institutes of Health, Bethesda, MD, USA, http://rsb.info.nih.gov/ij/). Before quantification of the artifact, both the original and MAR-applied CT images were converted into 8-bit image format thus pixel values were ranged from 0 to 255. Then, the images were binarized with threshold pixel value of 255^12^.

The binarized MAR-CT image was subtracted from the binarized original CT image. Then the area of artifact removed by the MAR algorithm was remained. The area was measured and recorded as the amount of the metal artefact (Fig. 1B).

### Statistical analysis

The mean value of the MAR-applied models deviation on each reference plane were obtained. The deviation of the 4 reference planes was compared using analysis of variance (ANOVA), while the 2 axial planes and 2 coronal planes were compared using the t-test. The inter-, intra-class correlation coefficients of the measurement values were evaluated with 95% confidence interval (CI). Pearson correlation analysis was performed to determine the relationship between model deviation and the amount of metal artifact. Statistical analyses of the data were performed using SPSS version 25.0 for Windows (IBM Corp. Armonk, NY, USA).

## Results

**Figure 3:**
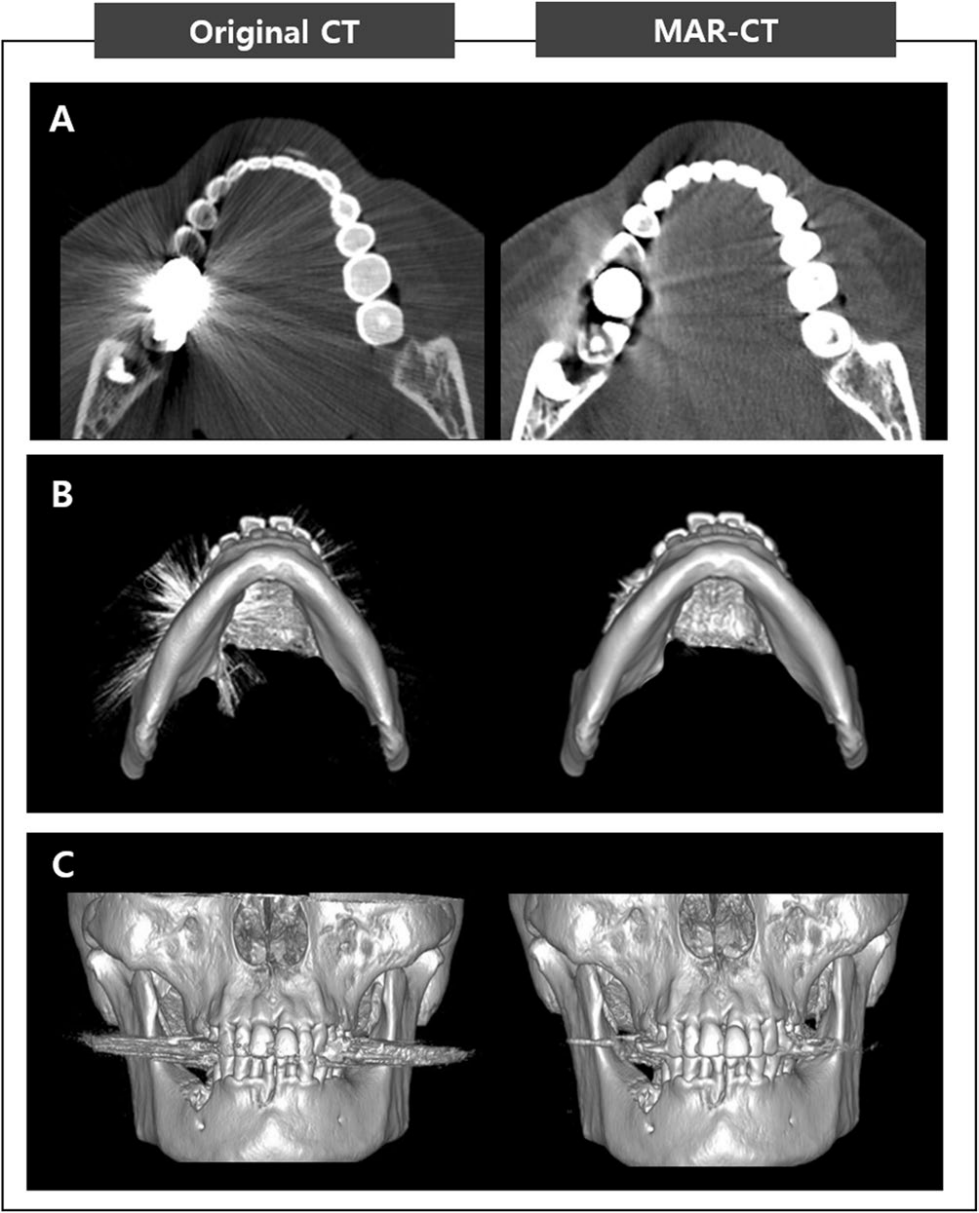
Original CT and MAR-applied CT. (**A**) Axial view, (**B**) volume rendering image (bottom view), (**C**) volume rendering image (frontal view). Note the significant reduction of metal artifacts in the MAR-CT image compared to the original CT. *MAR* metal artifact reduction.

**Figure 4:**
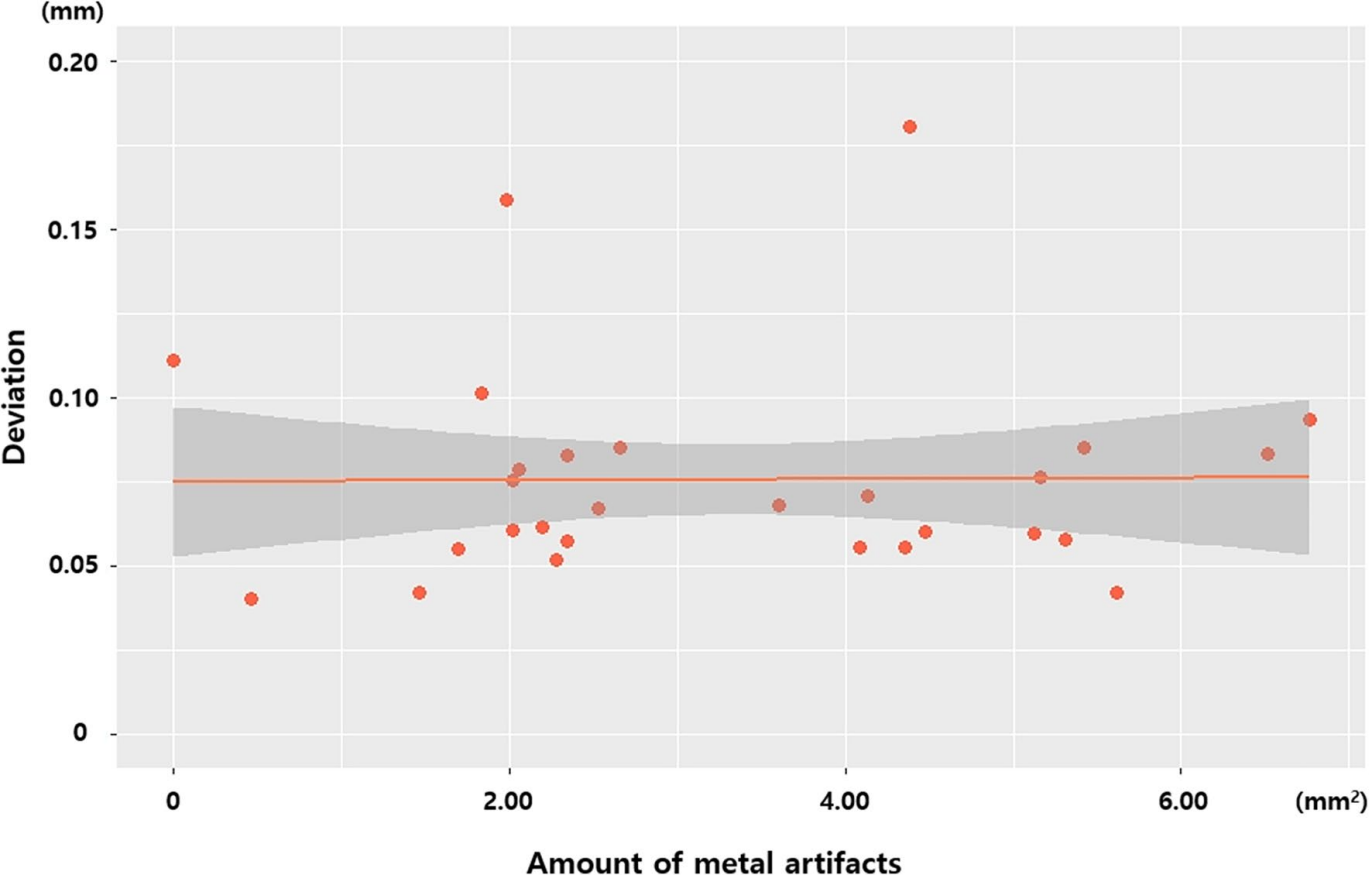
Scatter plot showing the correlation between the deviation (mm) of the MAR-applied model and the amount of metal artifact (mm^2^). No meaningful correlation is shown.

When compared the original CT with MAR-CT data, MAR algorithm effectively reduced metal artifact (Fig. 3). The mean value of metal artifact was 3.3654 mm^2^ and ranged from 0.0000 to 6.7630 mm^2^. The amount of artifact did not show significant correlation with the deviation of the MAR-applied digital model (r = 0.180, *p* = 0.342). The case with the largest amount of artifact showed a model deviation of 0.0900 mm, while that with the smallest artifact amount showed a deviation of 0.1100 mm (Fig. 4).

The overall mean deviation of the MAR-applied models compared to the corresponding original models was 0.0868 mm (range 0.0403–0.2648 mm). The deviation of each reference plane showed no significant difference (mean, axial maxilla = 0.0817; axial mandible = 0.0753; coronal right = 0.0998; coronal left = 0.0914). The deviation of the coronal planes was slightly higher tendency than that of the axial planes and the axial maxilla plane showed slightly larger deviation than the axial mandible plane. The right and left coronal planes presented the values with no statistically significant difference (Table 1). The intra- and inter-observer ICC values of deviation measurement were 0.934 (95% CI 0.897–0.958) and 0.910 (95% CI 0.873–0.934).

**Table 1:** Deviation of the digital models with metal artifact reduction algorithm (mean, standard deviation) and comparison of deviation according to the different reference planes.

Reference plane		Mean ± standard deviation (mm)	*P*-value*
Axial	Maxilla plane	0.0816 ± 0.0448	0.397
	Mandible plane	0.0753 ± 0.0560	
Coronal	Right plane	0.0988 ± 0.0945	
	Left plane	0.0914 ± 0.0577	
Total		0.0868 ± 0.0540	

^*^P < 0.05 was considered to indicate statistical significance, and the *P*-value was calculated using analysis of variance (ANOVA).

## Discussion

Currently, when creating a digital model from maxillofacial CT, white streaking artifact in image due to dental prosthesis appears as spicules around the dental arch unexceptionally. MAR algorithm, advanced considerably during recent years, reduces the artifact and as well as the effort of manual model manipulation by clinician, however, it causes overall deviation of CT data. Thus, for the first time, this study investigated the deviation level of dental digital models produced from MAR-applied CT data, as well as the correlation between the amount of metal artifact and the deviation.

In the current study, application of MAR did not lead to significant distortion of the digital models relative to the original models. Previous studies evaluated the accuracy of digital models produced using CT data obtained from different CT. One study reported that the mean deviation ranged from 0.44 to 0.77 mm in multi-detector CT, and from 0.165 to 0.386 mm with cone-beam CT^13^. In another study, Liang et al. reported that the mean deviation of digital model from CT data was 0.137 mm (range 0.165–0.638 mm) from cone-beam CT^14^, while von Wilmowsky et al., who studied cone-beam CT, reported 0.2676 mm of deviation^15^. The deviation found in the current study (0.0868 mm) was smaller than that reported in previous studies. It can be inferred that the deviation due to the application of MAR was lower than the deviations originated from different data sources.

Another important implication of this study was whether it is clinically acceptable. In the previous study, Hayashi et al. described that model with measurement accuracy up to 0.1 mm was clinically adequate for orthodontic diagnostic purpose^16^. Also, von Wilmowsky et al., reported that 0.2676 mm of deviation was thought to be clinically acceptable, as their digital model allowed a precise simulation of surgical procedures to benefit patients^15^. As a result of the current study, the deviation of the model due to the MAR application was relatively within the clinical acceptable level. Still, there were two cases showing deviation more than 0.1 mm, the error limitation suggested by one of the previous study. Therefore, the clinician should consider the purpose of the model and the possibility of model error when using MAR-applied CT based model.

Moreover, no significant differences in deviation values were found between different direction of plane. This confirmed that MAR application is feasible for maxillofacial digital modeling regardless of the specific area, in either the mandible or maxilla. Nonetheless, the deviation found for the maxilla was slightly larger than that found for the mandible. This may due to the fact that the maxilla is a more complex structure than the mandible. The MAR algorithm may cause more distortion on the delicate anatomical structures^13^.

Li et al. stated that the MAR algorithm altered the maximum HU values of CT data, thereby causing changes in the entire image data^8^. They reported that in some cases, application of the MAR algorithm to images with severe artifact resulted in incorrect modification of non-artifact area^8^. This tendency was shown in the current study as well. The model produced from the data showing ‘0 amount’ of artifact also revealed deviation from the original model when MAR algorithm applied. It can be presumed the subtle change of HU values and modification of non-artifact area due to MAR algorithm, may induce more influence on the delicate anatomic structure as maxillofacial region. Thus, even though this study reported the deviation was subtle when MAR-applied CT data was used for 3D model generation, it is important to be aware of the possibility of error occurring when dealing with any small complex anatomic structures.

An interesting result of this study was that the amount of metal artifact and the deviation of the 3D digital model showed no apparent correlation. Some previous studies have reported contrary results. According to Axente et al., the deviation of the image data increased as the number of prostheses increased when using the MAR option^17^. This discrepancy might be partially due to the use of different algorithm in the current study, which might explain the more reliable results. Another possibility for explaining these different results might be linked to differences in the experimental subjects. The previous study used round acrylic phantom with multiple metal rods inserted.

The current study was performed on actual patients with dental prostheses of various sizes and shapes. Differences among prostheses in size and shape may have major impacts on the resultant metal artifact creation^18^. Since the current study was conducted on image data with unpredictable metal artifact, with no clear trend, the results differed from those of previous studies performed using phantoms with a uniform shape. Therefore, it is necessary to conduct further in-depth research on the effect of MAR application and the distortion of STL model due to the distribution and the number of prosthesis overall in dental arch through a standardized quantification method.

Another limitation of this study was that only a single MAR algorithm was applied. As various types of MAR algorithms have been developed, a comparison of the accuracy of different algorithms would be a fruitful topic for further research. The reliability of these results would also be increased by a further analysis with a larger sample size of CT data, including a greater variety of dental prostheses.

In conclusion, MAR application of CT data presented a convenient method of 3D modeling, while maintaining a clinically applicable level of accuracy regardless of the different maxillofacial region. Furthermore, there was no significant difference in deviation depending on the amount of metal artifact, which closely related with the number of dental prostheses. Thus, it can be suggested that MAR algorithm may be used regardless of the dental prostheses for the quick and convenient manipulation of 3D digital models in clinical conditions.
